# Fumagillin inhibits growth of the enteric protozoan parasite *Entamoeba histolytica* by covalently binding to and selectively inhibiting methionine aminopeptidase 2

**DOI:** 10.1128/aac.00560-23

**Published:** 2023-10-24

**Authors:** Natsuki Watanabe, Yumiko Saito-Nakano, Naoaki Kurisawa, Keisuke Otomo, Kiyotake Suenaga, Kentaro Nakano, Tomoyoshi Nozaki

**Affiliations:** 1 Department of Biomedical Chemistry, Graduate School of Medicine, The University of Tokyo, Tokyo, Japan; 2 Department of Parasitology and Antimicrobial Resistance Research Center, National Institute of Infectious Diseases, Tokyo, Japan; 3 Department of Chemistry, Faculty of Science and Technology, Keio University, Kanagawa, Japan; 4 Degree Programs in Biology, Graduate School of Science and Technology, University of Tsukuba, Ibaraki, Japan; The Children's Hospital of Philadelphia, Philadelphia, Pennsylvania, USA

**Keywords:** *Entamoeba histolytica*, fumagillin, methionine aminopeptidase 2, patatin family

## Abstract

Amebiasis is an important cause of morbidity and mortality worldwide, and caused by infection with the protozoan parasite *Entamoeba histolytica*. Metronidazole is currently the first-line drug despite adverse effects and concerns on the emergence of drug resistance. Fumagillin, a fungal metabolite from *Aspergillus fumigatus,* and its structurally related natural and synthetic compounds have been previously explored as potential anti-angiogenesis inhibitors for cancers, anti-microbial, and anti-obese compounds. Although fumagillin was used for human amebiasis in clinical trials in 1950s, the mode of action of fumagillin remains elusive until now. In this report, we showed that fumagillin covalently binds to methionine aminopeptidase 2 (MetAP2) and non-covalently but abundantly binds to patatin family phospholipase A (PLA). Susceptibility against fumagillin of the amebic strains in which expression of *E. histolytica MetAP2* (*EhMetAP2*) gene was silenced increased compared to control strain. Conversely, overexpression of EhMetAP2 mutants that harbors amino acid substitutions responsible for resistance to ovalicin, a fumagillin analog, in human MetAP2, also resulted in decrease in fumagillin susceptibility. In contrast, neither gene silencing nor overexpression of *E. histolytica* PLA (EhPLA) affected fumagillin susceptibility. These data suggest that EhPLA is not essential and not the target of fumagillin for its amebicidal activity. Taken together, our data have demonstrated that EhMetAP2 is the primary target for amebicidal activity of fumagillin, and EhMetAP2 represents a rational explorable target for the development of alternative therapeutic agents against amebiasis.

## INTRODUCTION

*Entamoeba histolytica* causes human amoebiasis, which is the second leading cause of deaths among protozoan parasites in low- and middle-income countries. Approximately 50 million people, 0.6% of the global population, are infected and 10,000 infected are killed each year (1, 2). Metronidazole is the first-line drug of choice against amebic dysentery and liver abscesses. Metronidazole targets anaerobic energy metabolism, more specifically pyruvate: ferredoxin oxidoreductase, which is absent in humans. However, metronidazole therapy is sometimes associated with severe adverse effects such as neurotoxicity, optic and peripheral neuropathy, and encephalopathy, as well as mild adverse effects including headache, dizziness, abdominal pain, vomiting, fever, and depression (3). Therefore, new drugs with less adverse effects, particularly with novel mechanisms of action and structures are always needed.

Fumagillin, a secreted natural fungal metabolite, was first isolated from *Aspergillus fumigatus* (4). Fumagillin was previously utilized for the treatment of amebiasis patients early in the 1950s (5). In 1990s, fumagillin-related compounds, including TNP-470, were shown to possess angiogenesis inhibitory activity, thus were explored for chemotherapy of metastasized solid tumors until its derivatives with acceptable toxicity profiles reached clinical trials as anti-cancer agents (6, 7). Recently, fumagillin derivatives, including beloranib (8) and ZGN-1061, have been explored in human clinical trials in patients with obesity, including Prader-Willi syndrome, a genetic neurodevelopmental disorder, and type 2 diabetes (8).

The molecular target of fumagillin in humans was demonstrated to be methionine aminopeptidase 2 (MetAP2) by chemical validation. MetAP2 was found to be covalently and specifically bound to fumagillin and fumagillin derivatives from human cell lysates (9, 10). MetAP2 is engaged with the cotranslational removal of the initiator methionine residue from a nascent polypeptide, and this process is a prerequisite for the post-translational modifications at the amino terminus such as myristoylation and acetylation (11). The crystal structure of human MetAP2 and fumagillin demonstrated that a covalent bond is formed between a reactive epoxide of fumagillin and the highly conserved histidine residue at the 231 amino acid (a. a.) position located in the active site of human MetAP2 (12). However, in contrast to humans, the target of fumagillin in *E. histolytica* needs to be chemically and genetically demonstrated.

In this report, we demonstrated by the affinity pull-down approach using biotinylated fumagillin that fumagillin binds to MetAP2 and patatin family phospholipase A, PLA. Gene silencing and overexpression of *EhMetAP2* or *EhPLA* in *E. histolytica* showed that EhMetAP2, but not EhPLA, is associated with growth inhibition of the parasite by fumagillin and thus the major target of fumagillin in *E. histolytica*. These data are consistent with the fact that all amino acid residues implicated for the interaction of human MetAP2 with fumagillin are well conserved in *E. histolytica* MetAP2. Taken together, our chemical and genetic validation of EhMetAP2 as the authentic target of fumagillin should further facilitate derivatization of fumagillin to improve efficacy and safety for the development of new drugs against amebiasis.

## RESULTS

### Isolation of EhMetAP2 and phospholipase A as the target of fumagillin using biotinylated fumagillin

**Fig 1:**
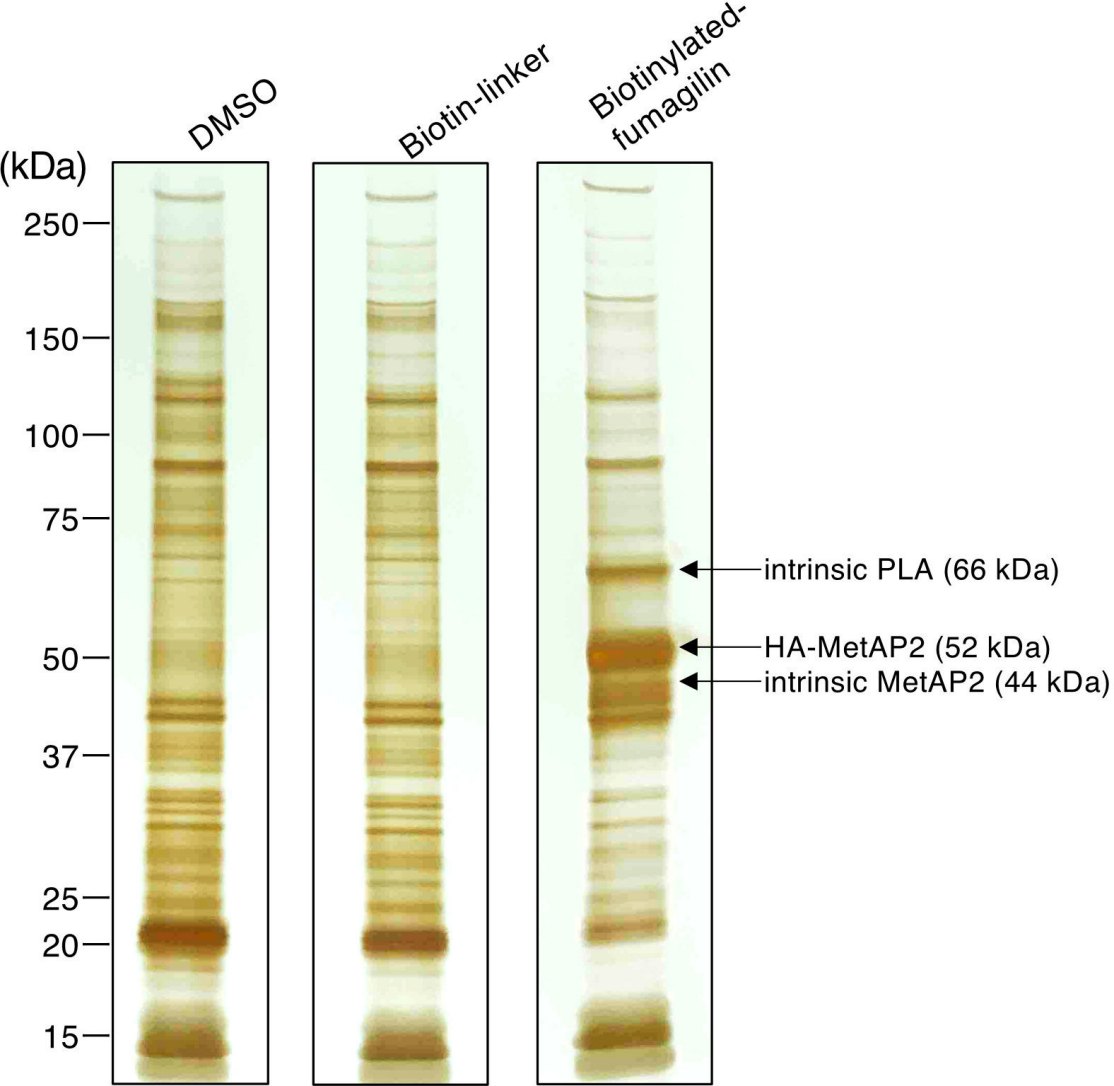
Isolation of fumagillin binding proteins in *E. histolytica*. The samples were prepared from lysates of HA-MetAP2 expressing strain, using biotinylated fumagillin. Control samples were also prepared in parallel using biotin-linker or DMSO only. The pull-down experiment was performed as described in Materials and Methods. Affinity purified samples were subjected to SDS-PAGE and silver stained. Approximately 10 µL of samples (20 µg equivalent) were loaded in each lane.

**Fig 2:**
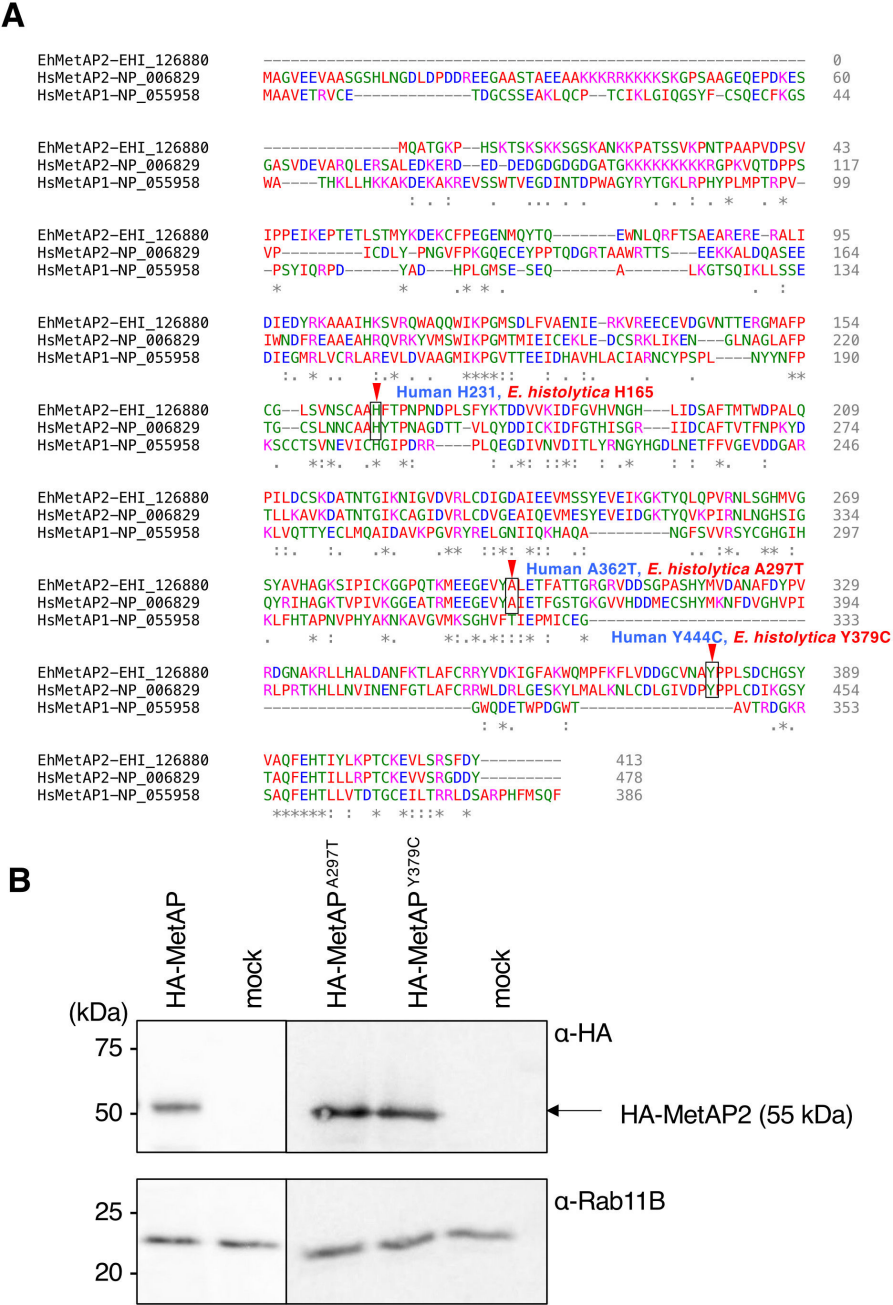
Comparison of *E. histolytica* MetAP2 and human MetAP1 and 2, and confirmation of expression of HA-EhMetAP2 wild type and mutants. (**A**) Amino acid alignment of *E. histolytica* MetAP2, human MetAP1, and MetAP2, created using ClustalOmega. The positions containing identical a. a. are marked with asterisks and those with conserved a. a. changes are marked with dots or colons. Three a. a. residues discussed in details in the text are marked with red arrows and labeled. (**B**) Immunoblot analysis of exogenously expressed EhMetAP2 wild type and mutants. Lysates from HA-MetAP2 wild type, HA-EhMetAP^A297T^, and HA-EhMetAP^Y379C^ expressing strains were subjected to SDS-PAGE and immunoblot analysis by anti-HA antibody. An arrow indicates 55 kDa bands corresponding to HA-MetAP2 wild type, HA-EhMetAP^A297T^, or HA-EhMetAP^Y379C^. Rab11B was used as an internal loading control.

To investigate the target of fumagillin in *E. histolytica*, affinity pull-down experiments using biotinylated fumagillin were conducted (Fig. 1; see Materials and Methods for organic synthesis of biotinylated fumagillin). The biotin moiety was incorporated into the sidechain of fumagillin at the C-4 position. We presumed that the biotin moiety unlikely interferes with binding to the target, based on the fact that potent fumagillin analogs such as TNP-470, which have a substitution at this position, retain their inhibitory activity (9, 10). Biotinylated fumagillin showed amebicidal activity with an IC_50_ value of 1.15 ± 0.03 µM, indicating that biotinylated fumagillin retains lower but reasonable inhibitory activity. For affinity isolation of fumagillin targets, we used the ameba line that expressed HA-tagged EhMetAP2 because we presumed that MetAP2 could be a potential binding protein of fumagillin. *E. histolytica* possesses only a single *MetAP2* gene (EHI_126880) but lacks *MetAP1* (AmoebaDB: http://amoebadb.org/amoeba/app, version 62)*,* while the human has single genes each encoding MetAP1 and MetAP2 (13). EhMetAP2 shows 46% or 23% a. a. identity to human MetAP2 and MetAP1, respectively. The biotinylated fumagillin was mixed and incubated with lysates from HA-EhMetAP2 expressing amoebas (for validation of expression of HA-tagged wild-type EhMetAP2, see the next section, Fig. 2.). The biotinylated fumagillin-bound proteins were enriched on avidin-conjugated beads and the proteins were separated by SDS-PAGE under reducing conditions. The proteins isolated with biotinylated fumagillin were visualized by silver staining (Fig. 1). Three bands, at approximately 66, 52, and 44 kDa, were exclusively detected by silver staining from the sample isolated with biotinylated fumagillin, but not from the sample with the control biotin linker and DMSO solvent. Detection of biotinylated fumagillin on the membranes with Avidin-HRP identified only 52 and 44 kDA bands but not 66, kDa band (Fig. S1). Immunoblot analysis of the samples with anti-HA antibody indicated that HA-EhMetAP2 was also detected as a 52 kDa band, which was enriched in the sample isolated with biotinylated fumagillin, compared to the sample isolated with the control biotin (Fig. S1A). These data are consistent with HA-EhMetAP2 was pulled down by biotinylated fumagillin (Fig. S1B). These results were consistent with the notion that 52 kDa HA-tagged EhMetAP2 and 44 kDa protein, which most likely corresponds to endogenous EhMetAP2, covalently bound to biotinylated fumagillin, whereas 66 kDa band of unknown identity was only reversibly associated with biotinylated fumagillin.

The affinity purified proteins with biotinylated fumagillin were analyzed by LC-MS/MS. A total of 174 proteins were identified, including 152 proteins identified from the sample isolated with biotinylated fumagillin, and 122 proteins identified from the biotin-liker control (Table S1). Ten most abundantly identified proteins with the highest quantitative values (QVs) are listed in Table 1. Top two hits, EhMetAP2 (EHI_126880) and phospholipase of the patatin family (EHI_200740), were detected exclusively from the biotinylated fumagillin sample but not from the control biotin-linker sample. EHI_200740, which is composed of 625 a. a. with the predicted molecular mass of 70.2 kDa, shows homology with patatin family phospholipases from *Dictyostelium discoideum* (XP_636632, 33% identity, e-value 2e-43) and *Anaerolineales bacterium* (MCC6187438, 35% identity, e-value 1e-40). Patatin from plant tissues was reported to possess phospholipase A_2_ and A_1_ activities (14). We therefore tentatively designated EHI_200740 as EhPLA. The QVs of these two proteins were similar (EhMetAP2: 37.157, EhPLA: 33.995) and significantly higher than that of other proteins (Table 1). Although more than 100 proteins were detected by pull-down experiments with biotinylated fumagillin, very small number of proteins with greater than twofold enrichment and the QV of >5 were obtained with biotinylated fumagillin compared to biotin-linker control (only two proteins: 40S ribosomal protein S7 and 60S ribosomal protein L27) (Table 1), suggesting interaction of fumagillin to EhMetAP2 and EhPLA is specific and other detected proteins should be regarded as background. Taken together, these data demonstrate that biotinylated fumagillin covalently binds to HA-EhMetAP2 and intrinsic EhMetAP2 (52 and 44 kDa, respectively), while it non-covalently binds to intrinsic EhPLA (66 kDa).

**TABLE 1:** The list of proteins affinity-purified with biotinylated fumagillin (top10)

Protein name	EHI number	Size	Biotinylated fumagillin (QV)	Biotin-linker (QV)	Fold enrichment^*a*^
Methionine aminopeptidase 2	EHI_126880	46 kDa	37.157	0	inf
Phospholipase patatin family protein	EHI_200740	70 kDa	33.995	0	inf
Grainin 1	EHI_167300	24 kDa	19.764	42.172	0.46
Cluster of 60S ribosomal protein L4 putative	EHI_035440	47 kDa	12.649	12.243	1.03
60S ribosomal protein L4 putative	EHI_035440	47 kDa	11.859	12.243	0.96
Actin	EHI_107290	42 kDa	10.277	10.883	0.94
Cluster of Elongation factor 1-alpha	EHI_052400	48 kDa	10.277	9.5226	1.07
Aldehyde-alcohol dehydrogenase	EHI_150490	96 kDa	10.277	6.8019	1.51
Elongation factor 1-alpha	EHI_052400	48 kDa	9.4868	9.5226	0.99
Peroxiredoxin	EHI_122310	27 kDa	9.4868	6.8019	1.39

^*a*^ Normalized relative ratio coprecipitated with biotin-fumagillin/biotin linker.

### Overexpression of EhMetAP2 mutant confers the resistance to fumagillin in *E. histolytica*

The identification of EhMetAP2 and EhPLA indicates that the target of fumagillin can be one or both of the two proteins. First, we investigated if EhMetAP2 is the target of fumagillin in a genetic approach. To genetically validate if the target of fumagillin is MetAP2 in *E. histolytica*, we created three ameba lines that overexpressed HA-tagged wild-type EhMetAP2, or one of two mutants, EhMetAP2^A297T^ or EhMetAP2^Y379C^. These EhMetAP2 mutants correspond to human MetAP2^A362T^ and MetAP2^Y444C^ mutants, respectively (Fig. 2A). These mutation in human MetAP2 were identified by screening of human MetAP2 mutations that can rescue the *Saccharomyces cerevisiae MetAP1* and *MetAP2* double knockout strain in the presence of fumagillin derivative, ovalicin (ovalicin-resistant human MetAP2) (13). Expression of HA-tagged wild-type EhMetAP2, EhMetAP2^A297T^, or EhMetAP2^Y379C^ in trophozoites under the tetracycline inducible system was validated by immunoblot analysis with anti-HA monoclonal antibody, showing a single band with an apparent molecular mass of 55 kDa (Fig. 2B). These data indicate that HA-EhMetAP2 wild type and mutants, with the expected molecular mass of 46 kDa plus 3 kDa, the latter of which corresponds to the 3 HA tag, shows an apparent molecular mass slightly larger than the predicted size. Expression of HA-EhMetAP2 wild type and mutants were robust and the levels of expression of EhMetAP2 were apparently comparable among the lines (Fig. 2B). Almost all transformant cells apparently expressed the HA-EhMetAP2 proteins (Fig. S2). Subcellular localization of HA-EhMetAP2 wild type and mutants examined by indirect immunofluorescence assay (IFA) with anti-HA antibody was mostly cytosolic (Fig. S2). We compared the IC_50_ values, the concentrations that show 50% growth inhibition of the parasite against the ameba lines, of fumagillin. The IC_50_ values of fumagillin against *E. histolytica* wild-type reference strain HM-1:IMSS cl6 was 69.0 ± 1.3 nM, which was slightly different from the mock-transfected control for tetracyclin-inducible expression (94.3 ± 7.9) (Table 2), which was about 70- to 90-fold lower than that of metronidazole (6.5 ± 0.3 µM) (15). An ameba line that expressed HA-EhMetAP^Y379C^ showed a 3.6-fold higher IC_50_ value compared to the mock transformant (*P*-value < 0.002) (Table 2). An amoeba line that overexpressed HA-tagged wild-type EhMetAP2 showed approximately twofold increase in the IC_50_ value (statistically not significant, *P* > 0.05). In contrast, expression of HA-EhMetAP2^A297T^ conferred only marginal resistance to fumagillin (not statistically significant, *P*-value > 0.05) (Table 2). This is in contrast to the previous report on human MetAP2 (13), and suggesting this a. a. residue may not be involved in the interaction with fumagillin.

**TABLE 2:** IC_50_ values of fumagillin against EhMetAP2- or EhPLA-overexpressing strains

Expression system	Protein overexpressed	IC_50_ values (nM)	*P* value against mock^*d*^
NA	None	69.0 ± 1.3	NA^*a*^
Constitutive	Mock^*b*^	49.1 ± 3.7	NA
	PLA	48.6 ± 2.5	0.9
Tetracycline-inducible	Mock^*c*^	94.3 ± 7.9	NA
	EhMetAP	201.2 ± 58.2	0.05
	EhMetAP-A297T	147.0 ± 44.1	0.09
	EhMetAP-Y379C	342.0 ± 33.4	**0.002**

^*a*^ Not applicable.

^*b*^ The constitutive expression plasmid, pEhEx-HA, was used to create mock control.

^*c*^ The inducible expression plasmid, pEhTex-HA, was used to create mock control.

^*d*^ Values in bold are statically significant (*P* < 0.05) by Fisher's exact test.

### Overexpression of EhPLA does not affect the sensitivity to fumagillin

**Fig 3:**
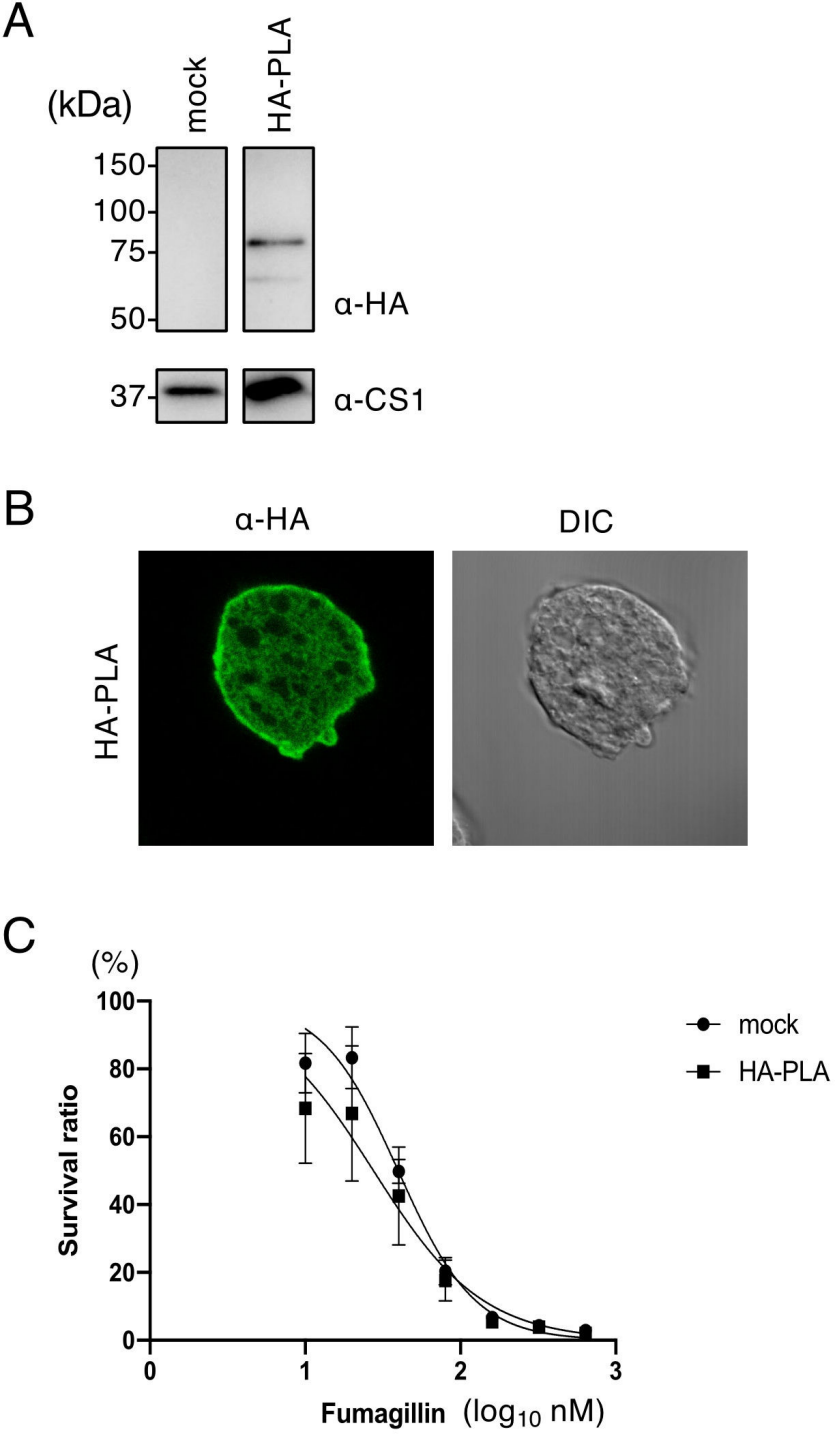
Expression of HA-EhPLA expressing strain and its fumagillin sensitivity. (**A**) Immunoblot analysis of HA- EhPLA. The expression of HA-EhPLA was confirmed by immunoblot using anti-HA antibody. One major 80 kDa band (EhPLA, 70 kDa and HA tag, 3 kDa) and a minor 60 kDa band were detected. Anti-CS antibody was used to ensure the equality of sample loading. (**B**) Localization of EhPLA examined by IFA. Fluorescence and differential interference contrast (DIC) images are shown. HA-EhPLA was detected by anti-HA antibody. (**C**) Growth inhibition of HA-EhPLA-expressing or mock strain by fumagillin. The percentage of live amebae after cultivation with fumagillin at various concentrations for 48 hours, as estimated by WST-1 assay, is shown. IC_50_ values calculated from this plot are shown in Table 2.

Overexpression of fumagillin target EhMetAP2 mutants conferred fumagillin resistance to *E. histolytica* (Table 2). To verify whether EhPLA is involved in fumagillin target or regulation of EhMetAP2 enzymatic activity, N-terminally HA-epitope tagged EhPLA-overexpressing transformant strain was established. The expression of HA-EhPLA in amoeba lysate was confirmed by immunoblot using anti-HA antibody (a 80 kDa band in Fig. 3A). The subcellular localization of HA-EhPLA, examined by IFA with anti-HA antibody, indicated that HA-EhPLA was strongly associated with the plasma membrane and weakly dispersed throughout the cytoplasm (Fig. 3B). Contrary to expectations, HA-EhPLA overexpressing strain did not affect fumagillin sensitivity compared to the control strain (The IC_50_ values of mock control and HA-EhPLA were 49.1 ± 3.7 and 48.6 ± 2.5 nM, respectively; *P*-value > 0.05) (Fig. 3C; Table 2).

### Fumagillin sensitivity increased by *EhMetAP2* gene silencing

**Fig 4:**
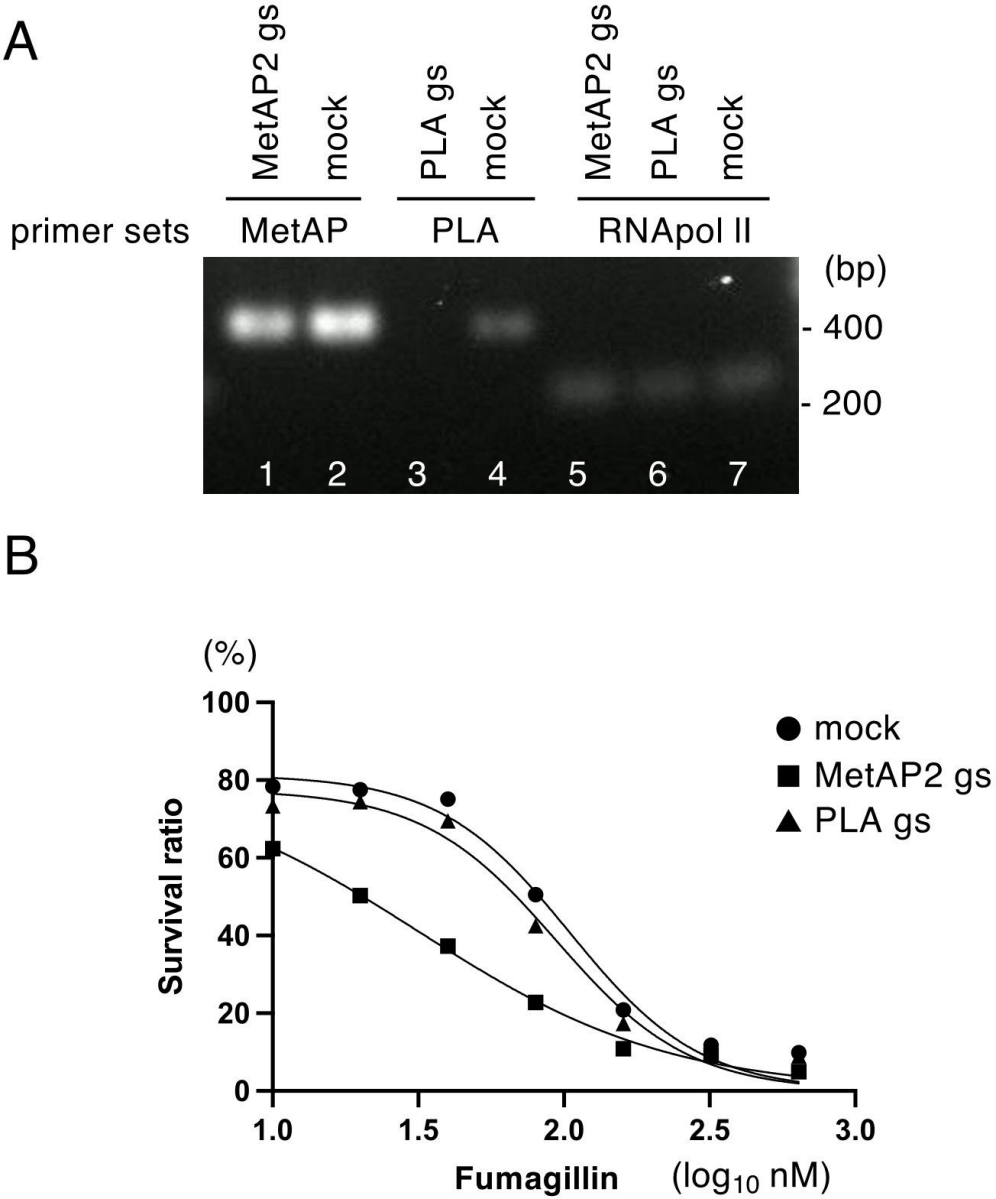
Validation of gene silencing of *EhMetAP2* and *EhPLA* genes and fumagillin sensitivity of the gene-silenced strains. (**A**) RT-PCR of *EhMetAP2* and *EhPLA* gene-silenced strains. cDNA was synthesized from mRNA of gene silenced and control strains. RT-PCR was conducted for *EhMetAP2* and *EhPLA* genes as well as *RNA pol II* (EHI_056690) gene as the internal control. PCR products were analyzed by agarose gel electrophoresis. Note approximately 50% of expression was suppressed in EhMetAP2gs strain, while in EhPLAgs strain, *EhPLA* expression was completely abolished. (**B**) Growth inhibition of EhMetAP2gs, EhPLAgs, and mock strains by fumagillin. The percentage of live amebae after cultivation with fumagillin at various concentrations for 48 hours, as estimated by WST-1 assay, is shown. IC_50_ values calculated from this plot are shown in Table 3. The representative plot, among triplicate experiments, was shown.

To further validate the premise that EhMetAP2 is the target of fumagillin, the ameba strain in which *EhMetAP2* was silenced by antisense small RNA-mediated transcriptional gene silencing was examined. The specific gene-silencing of *EhMetAP2* gene was confirmed by reverse transcriptase-polymerase chain reaction (RT-PCR) using cDNA (Fig. 4A). The expression level of *EhMetAP2* gene was decreased by approximately 50% in *EhMetAP2* gene-silenced strain (Fig. 4A). *EhMetAP2* gene-silenced strain showed growth defect in normal BI-S-33 medium (doubling time of *EhMetAP2* gene-silenced and mock strains, 48.6 ± 1.1 hours and 24.9 ± 3.2 hours, respectively: *P*-value = 0.0002). Due to chemical instability of fumagillin, the IC_50_ values of fumagillin against *EhMetAP2* gene-silenced and mock control strains varied among three sets of experiments; however, the trend was always consistent. *EhMetAP2* gene-silenced strain was more susceptible than the mock strain in all five trials (Fig. 4B). The IC_50_ values of fumagillin against *EhMetAP2* gene-silenced and mock control strains in the first three experiments were 60.8 ± 14.5 and 89.0 ± 11.9 nM, respectively (*P*-value = 0.2) (Table 3). In the fourth and fifth sets, *EhMetAP2* gene-silenced strain showed a lower IC_50_ value (29.5 ± 3.5 nM) than mock control (50.5 ± 2.1 nM) with statistical significance (*P*-value = 0.015) (Fig. S3).

**TABLE 3:** IC_50_ values of fumagillin against MetAP2- or PLA-gene-silenced strains

Gene silenced	IC_50_ values (nM)	*P* value against mock^*c*^
Mock^*a*^	89.0 ± 11.9	NA^*b*^
*EhMetAP2*	60.8 ± 14.5	0.2
*EhPLA*	90.9 ± 6.8	0.9

^*a*^ Empty pSAP plasmid was used to create mock control.

^*b*^ Not applicable.

^*c*^ Values in bold are statically significant (*P* < 0.05) by Fisher's exact test.

### Fumagillin inhibits EhMetAP2 enzymatic activity

Recombinant EhMetAP2 wild-type [glutathione S-transferase (GST)-EhMetAP2] proteins were expressed in the protease-deficient *S. cerevisiae* BY2777 strain and purified as a fusion protein with GST (16) (Fig. S4A). GST-EhMetAP2 was expressed as a 72 kDa fusion protein and purified at a ~95% purity based on the densitometric scanning of bands stained with Coomassie Brilliant Blue after SDS-PAGE (Fig. S4B). The GST-EhMetAP2 enzymatic assay was carried out as previously described (17) with some modification. The optimum pH for the GST-EhMetAP2 activity against the fluorogenic substrate L-methionine 4-methylcoumaryl-7-amide (Met-MCA) was pH 7.5 (Fig. S4C). The activity of GST-EhMetAP2 was reduced by 25% in the absence of MnCl_2_, or by more than 60% by the removal of MgCl_2_ or metal chelation with 10 mM ethylenediaminetetraacetic acid (EDTA), indicating the metal ions are required (Fig. S4D). The enzymatic turnover number *K*cat and Michaelis-Menten kinetics *K*m values of GST-EhMetAP2 for Met-MCA were 0.82 ± 0.07 per minute and 0.77 ± 0.04 mM, respectively. The inhibitory constant (*K*i) values, whose fumagillin concentration that decreased *V*max to 50% against GST-EhMetAP2, was 60 ± 33 nM (Fig. S4E).

### EhPLA is neither an essential gene in *E. histolytica* nor the target of fumagillin for the amebicidal effect

In order to verify whether EhPLA is also the target of fumagillin and to examine the possibility that EhPLA may be an accessory factor of EhMetAP2 in *E. histolytica*, the ameba strain in which *EhPLA* gene was silenced, was created like *EhMetAP2* gene-silenced strain described above. The expression of *EhPLA* gene was completely repressed in *EhPLA* gene-silenced strain (Fig. 4A). The expression of *RNA polymerase II* gene was not affected in either *EhMetAP2* or *EhPLA* gene-silenced strain (Fig. 4A). The fumagillin sensitivity was not reduced in *EhPLA* gene-silenced strain, when compared to mock control strain, unlike in *EhMetAP2* gene-silenced strain as shown above (Table 3). The IC_50_ values of *EhPLA* gene-silenced and mock control strains were 90.9 ± 6.8 and 89.0 ± 11.9 nM, respectively (*P*-value = 0.9) (Table 3; Fig. 4B).

Next, reciprocal immunoprecipitation assay of HA-MetAP2 with anti-HA antibody was conducted to confirm that EhPLA is not an effector of EhMetAP2. Mass spectrometric analysis of the immunoprecipitated sample showed that EhPLA was not detected as an HA-MetAP2 binding protein (Table S2). Taken together, these data indicate that *EhPLA* gene is not necessary for optimal growth under *in vitro* culture conditions, and thus, unlikely the target of fumagillin to be attributable for its amebicidal activity.

## DISCUSSION

### Redescription of fumagillin as a potent amebicidal compound that specifically targets EhMetAP2

In this study, we confirmed that fumagillin exhibits very potent amebicidal activity (the IC_50_ value: 49–94 nM) (Tables 2 and 3; Fig. S3). These values were influenced by the genetic background of *E. histolytica* strain and instability of fumagillin within solvent; however, fumagillin showed potency with ~130-fold lower IC_50_ than that of metronidazole (6.5 µM) and ~10-fold lower IC_50_ than auranofin (0.5 µM) (18) for the latter of which Phase I clinical trials to evaluate safety have been conducted (19). Fumagillin was previously utilized for the treatment of amebiasis patients early in the 1950s (5) and approved for the treatment of microsporidiosis in immunocompromised patients in France (20). Fumagillin was also shown to be efficacious against *Plasmodium falciparum* (21), *Cryptosporidium parvum* (17), and *Trichomonas vaginalis* (22). In 1990s, fumagillin-related compounds, including TNP-470, were shown to possess angiogenesis inhibitory activity, thus was explored for chemotherapy of metastasized solid tumors until its derivatives with low adverse effects reached clinical trials as anti-cancer agents (6, 7). Recently, fumagillin derivatives including beloranib (8) and ZGN-1061 (ZGN-1258) (23) have been explored in human clinical trials in patients with obesity, Prader-Willi syndrome, or type 2 diabetes (24). These studies indicate that those fumagillin derivatives are generally well tolerated with some adverse events of venous thromboembolism (25). Important safety issues that need to be precluded necessitate the in-depth understanding of the mechanism of action of the compound in both the human and pathogens.

In this study, we have demonstrated that the target of fumagillin in the important human enteric parasite *E. histolytica* is EhMetAP2 by both biochemical and reverse genetic approaches. Fumagillin binds to EhMetAP2 in an irreversible manner (Fig. S1), its amebicidal effect depends upon the amount of the target protein (Table 3), and enzyme inhibiting kinetics against GST-EhMetAP2 protein (*K*i values; 60 ± 33 nM) (Fig. S4) showed the comparable to IC_50_ value *in vitro* (69 ± 1.3 nM) (Table 2). The substrates of human MetAP2 were previously identified to be glyceraldehyde-3-phosphate dehydrogenase (GAPDH), nitric oxide synthase (eNOS), and cyclophilin A (26, 27). In this study, although the substrates of EhMetAP2 have not been identified, several surface proteins are predicted to be myristoylated (28), and can be potentially the candidates of EhMetAP2 substrates. In-depth structural understanding of EhMetAP2 and fumagillin in progress should further provide downstream strategy for more efficacious and safer drugs without adverse effects.

We have shown using biotinylated-fumagillin that fumagillin covalently binds to MetAP2 in *E. histolytica* (Fig. S1). Biotinylated fumagillin retained amebicidal activity with the IC_50_ value of 20 times higher than that of non-modified fumagillin. It should be noted that the IC_50_ of biotinylated fumagillin was still five times lower than that of metronidazole (15). It was also reported for human MetAP2 that biotinylated fumagillin bound to its target with 10-fold less affinity than TNP-470, a fumagillin derivative, but it retained potency (10). This premise was based on the previous observation in other organisms in the human, yeasts, *Plasmodium falciparum*, and *Giardia intestinalis* (10, 14, 21, 29, 30). It was shown that His231 in the active site of human, MetAP2 is irreversibly bound with the reactive epoxide on the cyclohexane ring of fumagillin (9, 12). This histidine is positioned in the metal center, and conserved in all known MetAPs including EhMetAP2 (Fig. 2A). Thus, we presume by analogy that this histidine is engaged in the covalent bond formation with the spiroepoxide on fumagillin.

### Dose response between EhMetAP2 and fumagillin sensitivity in *E. histolytica*

The sensitivity to fumagillin was clearly dependent on the amount of EhMetAP2 expressed in the trophozoites. Gene silencing of *EhMetAP2* increased the fumagillin sensitivity (Table 3; Fig. 4B; Fig. S3). In contrast, overexpression of EhMetAP2 conferred only marginal (statistically insignificant) decrease in fumagillin susceptibility. Furthermore, expression of EhMetAP2^Y379C^, which is equivalent to HsMetAP2^Y444C^, which was discovered by virtue of ovalicin resistance (13), increased fumagillin resistance by 3.6-fold (Table 2). Curiously, HsMetAP2^Y444C^ did not confer resistance to fumagillin in the yeast system (13). The Y444 residue is conserved in all MetAP2s but absent in MetAP1 (Fig. 2A), and involved in contact with the side chain of fumagillin (12). In human MetAP2, A362, also conserved among MetAP2s but not MetAP1 (Fig. 2A), is located in the β sheet partially forming the metal binding site, adjacent to E364, which is involved in metal coordination (12, 31). In contrast to HsMetAP2^Y444C^, HsMetAP2^A362T^ conferred resistance to both fumagillin and ovalicin (13). Our result showing lack of significant fumagillin resistance by EhMetAP2^A297T^ overexpression (Table 2) may indicate the structural difference of the metal-binding pocket. The observation may indicate that the structure of the fumagillin-binding pocket of EhMetAP2 might be different from that of human MetAP2, and suggest the possibility of developing highly selective fumagillin analogues against amebiasis.

### Patatin family PLA is a potential fumagillin target in *E. histolytica*, but unlikely responsible for amebicidal effect

We identified PLA as an unprecedent target of fumagillin-binding protein from the amebic lysate (Fig. 3). The quantitative values of EhMetAP2 and EhPLA by mass spectrometry were comparable (Table 1), suggesting that the number of EhPLA molecules bound to fumagillin was as high as EhMetAP2; alternatively, interaction of biotinylated fumagillin and EhPLA is as strong as that to EhMetAP2. Although EhPLA bound to and concentrated with biotinylated fumagillin was abundant and the identification EhPLA as a fumagillin binding protein was unequivocal, PLA is not covalently bound to fumagillin. Although the *Entamoeba* genome encodes five additional phospholipases of patatin family (EHI_073330, EHI_167000, EHI_060610, EHI_153390, and EHI_068060), none of these patatin family phospholipases were identified from biotinylated fumagillin affinity experiments (Table 1), ensuring the specific interaction between EhPLA and biotinylated fumagillin. As MetAP2 is the only protein that was identified by affinity pulldown with biotinylated fumagillin from the human cell lysate (9, 10), the identification of EhPLA with biotinylated fumagillin indicates *Entamoeba*-specific interaction.

However, EhPLA does not seem to be involved in amebicidal effects of fumagillin. Neither overexpression nor gene silencing of *EhPLA* did not affect growth of trophozoites in the regular BI-S-33 medium, suggesting EhPLA is not essential *in vitro*. Furthermore, neither overexpression nor gene silencing of *EhPLA* affected sensitivity against fumagillin (Fig. 3 and 4; Table 3). Further analysis is needed to better understand whether fumagillin directly or indirectly binds to EhPLA, or if directly, how EhPLA interacts with the acyl chain of fumagillin. Patatin is originally identified as potato storage protein, and displays lipid acyl hydrolase, acyl transferase activities (32), and both PLA_2_ and PLA_1_ activities (14). Proteins encoding patatin-like domains are ubiquitously conserved among eukaryotes and prokaryotes (33). The biological roles of patatin family phospholipases are broad. In animal cells, cytosolic PLA_2_ cleaves an *sn*-2 ester bond of phospholipids to release, typically, arachidonic acid and lysophospholipids, which act on second messengers for various downstream cellular responses (34). In addition, human patatin family phospholipases are known to be involved in diverse lipid metabolism, such as lipolysis, lipogenesis, and immune regulation (35). In contrast, plant patatin phospholipases possess lipid acyl hydrolase activity of patatin and are important for the rapid degradation of cell membranes for signaling and defense against virus infection (36, 37). Apicomplexan parasites *Plasmodium* species cannot synthesize fatty acids and cholesterol *de novo*, and thus scavenge phospholipids from the erythrocyte membranes and human serum (38, 39). One of the patatin family phospholipases in *Plasmodium* has been shown to be involved in differentiation, called gametogenesis, which takes place in the mosquito midgut, and essential for malaria transmission (40). The specific substrates and products of EhPLA need to be clarified in future to understand the fate of fumagillin binding to EhPLA. One should also note that our demonstration of PLA as a stable and abundant binding protein for fumagillin in the ameba casts a slight doubt that MetAP2 is a single target of fumagillin in humans.

## MATERIALS AND METHODS

### Cells and reagents

Trophozoites of *E. histolytica* strains HM-1:IMSS cl6 (41) and G3 (42) were axenically maintained in BI-S-33 medium (BIS) at 35.5°C as previously described (41). The anti-HA (clone 16B12) monoclonal antibodies were purchased from Covance (Princeton, NJ, USA). The production of rabbit polyclonal antibodies against EhCS1 and Rab11B were previously described (40, 41).

### General experimental procedures of the synthesis of chemical products

Optical rotations were measured with a JASCO DIP-1000 polarimeter. IR spectra were recorded on a Bruker ALPHA instrument. All NMR spectral data were recorded on a JEOL JNM-ECS400 spectrometer for ^1^H (400 MHz) and ^13^C (100 MHz). ^1^H NMR chemical shifts (referenced to residual CD_3_OD observed at *δ*_H_ 3.31) were assigned using a combination of data from COSY and HMQC experiments. Similarly, ^13^C NMR chemical shifts (referenced to CD_3_OD observed at *δ*_C_ 49.0) were assigned based on HMBC and HMQC experiments. HRESIMS spectra were obtained on a Waters LCT Premier XE time-of-flight (TOF) mass spectrometer. Chromatographic analyses were performed using an HPLC system consisting of a pump (model PU-2080, JASCO) and a UV detector (model UV-2075, JASCO). All chemicals and solvents used in this study were the best grade available and obtained from a commercial source (Nacalai Tesque). All moisture-sensitive reactions were performed under an atmosphere of argon or nitrogen, and the starting materials were azeotropically dried with toluene before use. Reactions were monitored by thin-layer chromatography (TLC), and TLC plates were visualized by both UV detection and phosphomolybdic acid solution. Silica Gel 60N (Irregular, 63–212 μm) were used for open column chromatography unless otherwise noted.

### Plasmid construction

The full-length protein coding region of *EhMetAP2* gene (EHI_126880) was amplified by PCR with following primers 5′-CTTATCCATATGATGTTCCAGATTATCCCGGG ATGCAAGCTACGGGAAA-3′ and 5′-TTAAGTTTAAAAAAGAAGAGTTCAACTCGAGTTAATAATCAAAACTTCTTGAAAG-3′ and inserted into SmaI and XhoI sites of pEhTex-HA to express EhMetAP2 protein fused with the three tandem hemagglutinin (HA) repeats at the amino terminus under the inducible tetracycline-dependent promoter (43). In-Fusion HD Cloning Kit (Clontech Laboratories, CA, USA) was used to construct the plasmid pEhTex-HA-EhMetAP2.

Plasmids that contain the protein-coding region of mutated EhMetAP2, EhMetAP2^A297T^ and EhMetAP2^Y379C^ were created using pEhTex-HA-EhMetAP2 with PCR-based site directed mutagenesis using Prime STAR Mutagenesis Basal Kit (Takara Bio).

A plasmid to establish *E. histolytica* lines that express EhPLA (EHI_200740) fusion protein containing HA-tagged at the amino terminus was constructed as follows. The full-length protein coding region of *EhPLA* gene was amplified by PCR with following primers: 5′-TCGAGACCGAGGAGAGGGTTAGGGATAG-3′ and 5′-GAACCCGGGATGAATATAAACCAACAACAAGAC-3′, where the restriction enzyme sites are underlined. PCR-amplified fragments were digested by XmaI and XhoI, and ligated into pEhEx-HA (44) that were predigested with the two enzymes to construct the plasmid. To construct plasmids for gene silencing of *EhMetAP2* and *EhPLA* genes, initial 420 bp fragments of the *EhMetAP2* and *EhPLA* coding region were PCR amplified with the following primers: 5′-GAAAGGCCTATGCAAGCTACGGGAAAACCAC-3′ and 5′- GAAGAGCTCTTCACATTCTTCTCTAACTTTTCTC-3′ (*EhMetAP2*); 5′- GAAAGGCCTATGAATATAAACCAACAACAAGAC −3′ and 5′- GAAGAGCTCTGGATATTTAGCAATAACTC −3′ (*EhPLA*). These PCR fragments were digested by StuI and SacI, and ligated into StuI- and SacI-double digested psAP2-Gunma (45, 46). These plasmids were designated as psAP2-MetAP2 and psAP2-PLA.

### Establishment of *E. histolytica* transformants

To establish overexpressing *E. histolytica* strains, the trophozoites of HM-1:IMSS cl6 were transfected with pEhExHA-MetAP2, pEhExHA-PLA, or pEhExHA by lipofection as previously described (45). To establish *E. histolytica* gene-silenced strains, the trophozoites of G3 strain were transfected with psAP2-MetAP2, psAP2-PLA, or psAP2-Gunma by lipofection. G3 strain was previously created by Prof. David Mirelman’s group by introducing a plasmid that contains the upstream region of *amebapore A* gene promoter (47), and was previously used in a number of our studies (48, 49) in which gene of interest were specifically silenced by introducing the modified plasmid that we created, psAP2-Gunma and its derivatives (47, 50), and contain a fragment of the first ~420 bp of the target genes. Geneticin was added at a concentration of 1 µg/mL at 24 hours after transfection, and the geneticin concentrations were gradually increased until it reached 6–10 µg/mL in next 2 to 3 weeks. To induce expression of HA-tagged EhMetAP2 wild type and mutants, the transformants were cultured in the presence of 10 µg/mL tetracycline for 18 hours.

### Immunoblot analysis

Approximately 10^5^ trophozoites were harvested in the exponential growth phase, washed twice with phosphate buffered saline (PBS) pH 7.4, and resuspended in 50 µL of lysis buffer (50 mM Tris-HCl, pH 7.5, 150 mM NaCl, 1% Triton X-100) containing 50 µg/mL of E-64, and Complete mini protease inhibitor cocktail (Roche). Approximately 20 µg of the total cell lysates were separated on 12% SDS-polyacrylamide gels and subsequently electro transferred onto nitrocellulose membranes. The membranes were incubated with 5% non-fat dried milk in TBS-T (50 mM Tri-HCl, pH8.0, 150 mM NaCl, and 0.05% Tween-20) for 30 minutes. The proteins were reacted with anti-HA mouse IgG (with the dilution of 1:1,000), rabbit antiserum against CS1 (1:1,000) or Rab11B (1:500) at 4°C overnight. After the reaction with the primary antibodies, the membranes were washed with TBS-T three times. The membranes were further reacted with HRP-conjugated anti-mouse (anti-HA) or anti-rabbit (anti-CS1, anti-Rab11B) IgG antiserum (1:6,000 or 1:8,000, respectively) at room temperature for 1 hour. After washing with TBS-T three times, the specific proteins were visualized with chemiluminescence detection using Immobilon Western Chemiluminescent HRP Substrate (Millipore Corporation, MA, USA) according to the manufacturer’s protocol.

### Determination of the 50% inhibitory concentrations of fumagillin resistance assay

Approximately 5 × 10^3^ *E. histolytica* trophozoites were incubated in 280 µL of BI-S-33 medium containing a serial dilution of fumagillin, metronidazole, or only dimethyl sulfoxide (DMSO) in a well on a 96-well plate at 35.5°C for 48 hours. The concentrations of fumagillin and metronidazole ranged 10–640 nM and 0.5–32 µM, respectively. After incubation, BI-S-33 medium was removed and 100 µL of WST-1 working solution (a mixture of WST-1 and OPTI-MEM with the ratio of 1:9 in volume). After the plate was incubated at 37°C for 30 minutes, the absorbance at 450 nm was measured.

### Synthesis of biotinylated fumagillin

**Fig 5:**
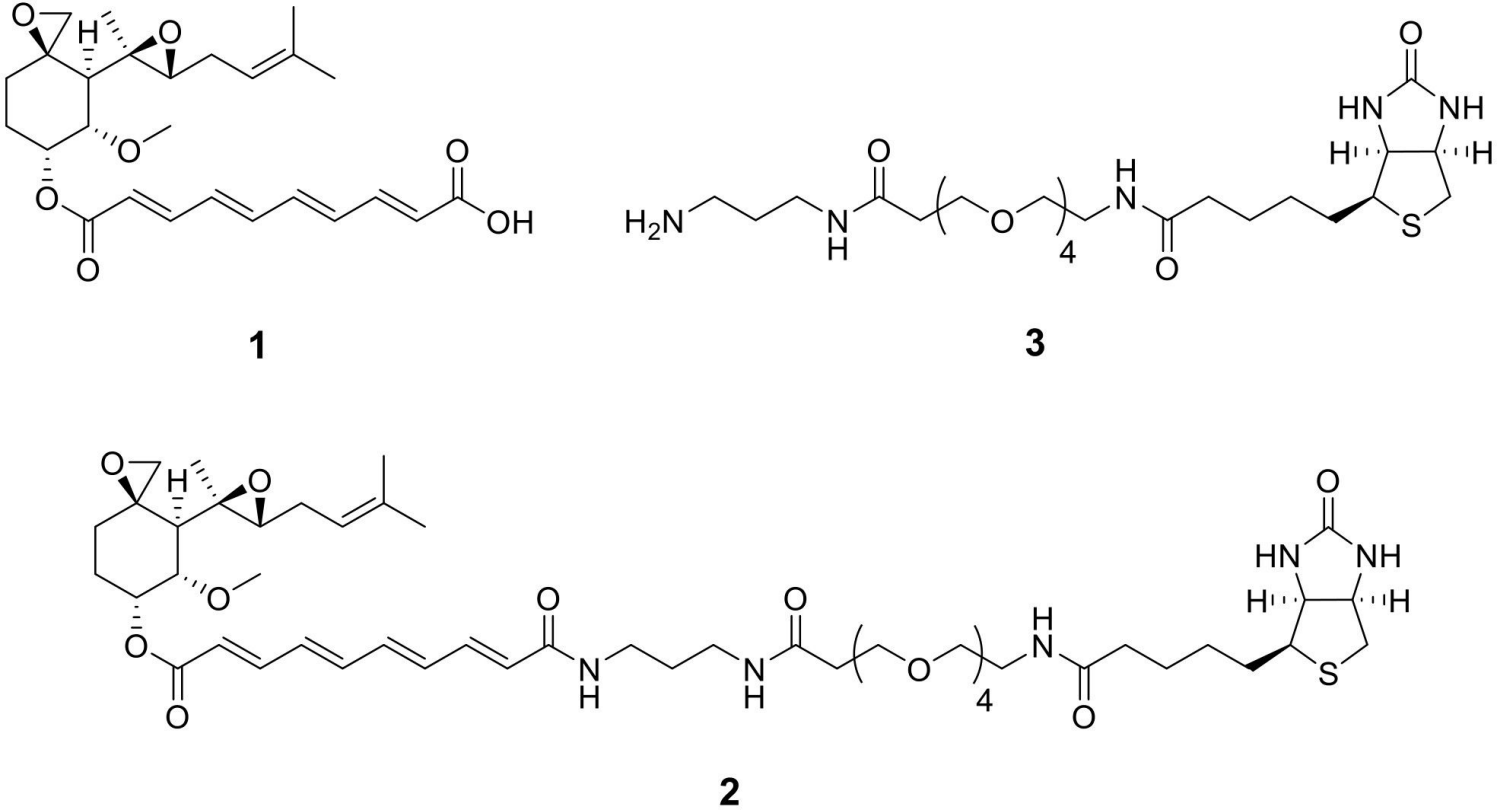
Structures of fumagillin (1), biotinylated fumagillin (2) and biotin-linker (3).

To identify the target of fumagillin, biotinylated fumagillin was synthesized as follows (Fig. 5).

***N-(3-aminopropyl)-1-{5-[(3aS,4S,6aR)-2-oxohexahydro-1H-thieno(3,4-d)imidazol-4-yl]pentanamido}-3,6,9,12-tetraoxapentadecan-15-amide* (biotin-linker, 3):** to a solution of known azide (51) (30.0 mg, 0.048 mmol) in EtOH (2 mL) was added 20% Pd(OH)_2_/C (50% water content, 10 mg). The reaction mixture vigorously stirred under a hydrogen atmosphere at room temperature for 3 hours. The reaction mixture was filtered, and the filtrate was concentrated to give the crude amine 3 (29.0 mg), which was used for the next reaction without further purification.

***(3R,4S,5S,6R)−5-methoxy-4-[(2R,3R)−2-methyl-3-(3-methylbut-2-en-1-yl)oxiran-2-yl]−1-oxaspiro(2.5)octan-6-yl (28E,30E,32E,34E)−5,21,27-trioxo-1-[(3aS,4S,6aR)−2-oxohexahydro-1H-thieno(3,4-d)imidazol-4-yl]−9,12,15,18-tetraoxa-6,22,26-triazahexatriaconta-28,30,32,34-tetraen-36-oate* (biotinylated fumagillin, 2):** to a stirred solution of the fumagillin dicyclohexylamine salt (60% purity, SINOVA, #SL-09704, 8.0 mg, equivalent to 0.0075 mmol) and a part of amine 3 (10.3 mg) in DMF (0.1 mL) cooled at 0°C were added DIPEA (one drop) and HATU (7.1 mg, 0.019 mmol). After stirring at room temperature for 1 hour, the reaction mixture was diluted with 10% aqueous citric acid (1 mL) and extracted with CH_2_Cl_2_ (10 mL × 3). The combined organic layers were washed with saturated aqueous NaHCO_3_ (10 mL) and brine (10 mL), dried over anhydrous Na_2_SO_4_, and concentrated. The residue was purified by HPLC [Cosmosil 5C_18_-MS-II (20 × 250 mm); flow rate 5 mL/minute; detection at 300 nm; solvent 72% MeOH] to give biotinylated fumagillin (2) (5.3 mg, *t*_R_ = 36.7min, 0.0054 mmol, 72% yield converted from the purity) as a colorless oil; (α)_D_^25^ +11 (*c* 0.44, MeOH); IR (neat) 3291, 3078, 2928, 2872, 1699, 1652, 1622, 1543, 1456, 1353, 1326, 1301, 1235, 1171, 1127 cm^-1; 1^H NMR (400 MHz, CD_3_OD, Fig. S5) *δ* 7.38 (dd, *J* = 15.4, 11.3 Hz, 1H), 7.22 (dd, *J* = 15.0, 10.9 Hz, 1H), 6.82–6.67 (m, 2H), 6.63–6.55 (m, 2H), 6.11 (d, *J* = 15.0Hz, 1H), 6.04 (d, *J* = 15.4 Hz, 1H), 5.71 (m, 1H), 5.25 (m, 1H), 4.49 (dd, *J* = 8.0, 4.8Hz, 1H), 4.30 (dd, *J* = 8.0, 4.4 Hz, 1H), 3.73 (dd, *J* = 11.3, 3.0 Hz, 1H), 3.73 (t, *J* = 6.3Hz, 2H), 3.65–3.59 (m, 12H), 3.53 (t, *J* = 5.4 Hz, 2H), 3.42 (s, 3H), 3.35 (t, *J* = 5.4 Hz, 2H), 3.30 (m, 1H), 3.24 (t, *J* = 6.9 Hz, 2H), 3.22 (m, 1H), 2.99 (d, *J* = 4.5Hz, 1H), 2.92 (dd, *J* = 12.9, 4.8 Hz, 1H), 2.72–2.68 (m, 2H), 2.59 (d, *J* = 4.5 Hz, 1H), 2.45 (t, *J* = 6.1 Hz, 2H), 2.33 (m, 1H), 2.22 (m, 1H), 2.22 (t, *J* = 7.5 Hz, 2H), 2.15 (ddd, *J* = 13.6, 8.4 4.5 Hz, 1H), 1.98 (d, *J* = 11.3 Hz, 1H), 1.96–1.83 (m, 2H), 1.78–1.54 (m, 6H), 1.76 (brs, 3H), 1.68 (brs, 3H), 1.48–1.39 (m, 2H), 1.21 (s, 3H), 1.09 (ddd, *J* = 13.6, 3.2, 2.4 Hz, 1H); ^13^C{^1^H} NMR (100 MHz, CD_3_OD, Fig. S4) *δ* 176.1, 174.1, 168.5, 167.8, 166.1, 145.7, 141.3, 141.0, 139.4, 136.0, 135.5, 134.2, 126.8, 123.2, 119.8, 80.8, 71.6 (3C), 71.5, 71.5, 71.4, 71.3, 70.6, 68.3, 68.0, 63.4, 62.4, 61.6, 60.6, 60.5, 57.0, 56.9, 51.7, 41.1, 40.4, 38.0, 37.9, 37.8, 36.7, 30.3, 30.2, 29.8, 29.5, 28.3, 26.9, 26.6, 25.9, 18.1, 14.2; HRMS (ESI-TOF) *m/z* 1010.5165 [M + Na]^+^ (calculated for C_50_H_77_N_5_O_13_SNa 1010.5136).

### Purification and identification of binding proteins of biotinylated fumagillin

Approximately 6 × 10^6^ trophozoites of the HA-EhMetAP2 expressing amoeba strain were cultured on a 10 cm diameter dish in BIS medium under the anaerobic conditions using Anaerocult (Merck, Darmstadt, Germany). The amoebae were detached from the dishes by adding cold PBS and incubated on ice for 10 minutes. The cells were collected by centrifugation, lysed with 800 µL of lysis buffer, and used in immunoblot analysis. After the insoluble debris was removed by centrifugation at 16,000 × *g* at 4°C for 5 minutes, the lysate (“total lysate”) was mixed and incubated with approximately 50 µL of Pierce NewtrAvidin agarose beads (50% slurry) (Thermo Fisher Scientific, Waltham, MA) 4°C for 1 hour to reduce non-specific binding in immunoprecipitation. After centrifugation at 3,000 × *g* at 4°C for 5 minutes, the supernatant was transferred to a new 1.5 mL tube and 1.74 µM biotinylated fumagillin or biotin-linker was added to the tube, and the mixture was incubated at 4°C for 2 hours. The concentration of biotinylated fumagillin was 8.6-fold higher than the concentration of IC_50_ value of HA-EhMetAP2 cells. Approximately 50 µL of NewtrAvidin agarose beads was added to and incubated with the soluble fraction at 4°C for 2 hours. The soluble unbound fraction was collected after centrifugation at 3,000 × *g* at 4°C for 3 minutes (“unbound fraction”). The agarose beads collected by centrifugation were washed three times with 1 mL of lysis buffer and eluted with 50 µL of 2 × SDS sample buffer [0.25 M Tris-Hcl(pH 6.8), 8% SDS, 8% 2-mercaptoethanol, and 40% glycerol, 0.004% bromophenol blue] by incubating the tube in boiling water for 5 minutes. Each sample was separated using SDS-PAGE, and visualized by silver staining. The in-gel trypsin digestion of proteins, liquid chromatography, and time-of-flight tandem mass spectrometry (LC-ToF MS/MS) were performed at Mass Spectrometry and Proteomics Facility in Biological Chemistry, School of Medicine of Johns Hopkins University. Proteins were digested with trypsin, labeled using the eight-plex iTRAQ isobaric mass tags (ABSciex) and analyzed by tandem mass spectrometry on an LTRQ Vwlos Orbitrap interfaced with an Eksigent 2D NanoLC (52).

### Immunofluorescence assay

Approximately 5 × 10^3^ *E. histolytica* trophozoites were incubated in 50 µL BI-S-33 medium in 8 mm round wells on a slide glass at 35.5°C for 15 minutes. Cells were fixed with 3.7% paraformaldehyde and subsequently permeabilized with 0.2% saponin in PBS containing 1% bovine serum albumin for 10 minutes each at room temperature. The cells were reacted with anti-HA mouse IgG (with the dilution of 1:1,000). After washing with PBS three times, the cells were reacted with Alexa Fluor-488 anti-mouse IgG secondary antibody (1:1,000). The samples were observed using Carl Zeiss LSM780 Meta laser-scanning confocal microscope. The resultant images were further analyzed using Zen software (Carl Zeiss, Oberkochen, Germany).

### Immunoprecipitation of HA-PLA

Approximately 6 × 10^6^ trophozoites of the HA-EhMetAP2 expressing amoeba strain were cultured on a 10 cm diameter dish in BIS medium under the anaerobic conditions using Anaerocult (Merck, Darmstadt, Germany). The amoebae were detached from the dishes by adding cold PBS and incubated on ice for 10 minutes. After centrifugation at 800 × *g* at 4°C for 3 minutes to collect the cells, the supernatant was removed and the amoebae were resuspended in 500 µL of 8 mg/mL dithiobis (succinimidyl propionate) (DSP) solution (Thermo Fisher, MA, USA). The mixture was incubated on the rotator (10 rpm) at 4°C for 30 minutes. To quench the reaction, 50 µL of 1 M Tris-HCl, pH 7.5 was added and the mixture was further incubated as above for 10 minutes. After the amoebae were treated with DSP, they were washed with PBS. The cells were lysed with 800 µL of lysis buffer, and the insoluble debris was removed by centrifugation at 16,000 × *g* at 4°C for 5 minutes, the lysate (“total lysate”) was mixed and incubated with approximately 50 µL of Protein G Sepharose beads (GE Healthcare, IL, USA) (80% slurry) 4°C for 1 hour to reduce non-specific binding in immunoprecipitation. After centrifugation at 3,000 × *g* at 4°C for 5 minutes, the supernatant was transferred to a new 1.5 mL tube containing approximately 50 µL (80% slurry) of anti-HA monoclonal antibody produced in mouse, clone HA-7, purified immunoglobulin conjugated to agarose beads (Sigma Aldrich). The tubes were incubated at 4°C for 3.5 hours. The soluble unbound fraction was collected after centrifugation at 3,000 × *g* at 4°C for 3 minutes (“unbound fraction”). The agarose beads collected by centrifugation were washed three times with 1 mL of lysis buffer and eluted with 50 µL of 2 × SDS sample buffer [0.25 M Tris-Hcl (pH 6.8), 8% SDS, 8% 2-mercaptoethanol, 40% glycerol, and 0.004% bromophenol blue] by incubating the tube in boiling water for 5 minutes. Each sample was separated using SDS-PAGE, and visualized by silver staining. The in-gel trypsin digestion of proteins, liquid chromatography, and time-of-flight tandem mass spectrometry (LC-ToF MS/MS) were performed at Mass Spectrometry and Proteomics Facility in Biological Chemistry, School of Medicine of Johns Hopkins University as described above.

### Expression and purification of recombinant EhMetAP2 in yeast system

A synthetic gene coding for EhMetAP2 with *Saccharomyces cerevisiae* codon was synthesized (Eurofin genomics) and inserted into EcoR1-Xho1 sites of the yeast expression vector pYEX4T-1 (Clontech) to express N-terminus GST-fusion protein. The plasmid was introduced into protease deficient *S. cerevisiae* BY2777 (*MATa prb1-1122 prc1-407 pep4-3 ura3-52 leu2 trp1*) (16), which was provided by the National Bio-Resource Project (NBRP) of the MEXT, Japan, and selected with leucine minus minimal medium at 30°C. Expression of GST-EhMetAP2 was induced by the addition of 5 µM CuSO_4_ for 18 hours at 30°C. Two hundred milliliters of culture cell pellet was broken by the glass beads with 5 mL lysis buffer (50 mM Tris, pH 6.8, 150 mM NaCl, 0.1 mM MnCl_2_, 0.1 mM MgCl_2_, 1 mM PMSF). Cell lysate obtained after the 13,000 × *g* centrifugation for 10 minutes was incubated with 500 µL glutathione 4B-Sepharose resin to immobilize the GST-EhMetAP2 protein for 18 hours at 4°C (53). After the resins were extensively washed with lysis buffer, the GST-EhMetAP2 protein was eluted by 200 µL elution buffer [100 mM reduced glutathione (GSH), 50 mM Tris, pH 8.0, 0.1 mM MnCl_2_, 0.1 mM MgCl_2_]. GST-EhMetAP2 was concentrated with Microcon-10 kDa (Millipore) with 2 mL of elution buffer to remove the excess GSH in the eluted sample. Purified GST-EhMetAP2 protein was mixed with final 10% glycerol, and stored under −80°C.

### Methionine aminopeptidase enzyme assay

The aminopeptidase activity was assayed fluorometrically based on hydrolysis of L-methionine 4-methycoumaryl-7-amide (Met-MCA; Peptide Institute, Inc., Japan) in a 96-well microplate format (17). In brief, 4 pmol of GST-EhMetAP2 protein was reacted with 0.25–2.0 mM Met-MCA (Code 3149-v: Peptide Institute, Inc, Japan) containing 50 mM Tris-HCl (pH 7.5), 1 mM MgCl_2_, 1 mM MnCl_2_, 0.01% Triton X-100 in 20 µL reaction, corresponding to the final concentration of EhMetAP was 200 nM. The fluorescence release of 7-AMC was measured after 120 minutes incubation at 37°C at an excitation wavelength of 370 nm and an emission wavelength of 465 nm with a DTX880 Multimode Detector (Beckman Coulter, Brea, CA, USA) (54). The Michaelis constant (*K*m) was determined by the end point assay. In the fumagillin inhibition assay, varying concentration of substrate Met-MCA and recombinant MetAP was pre-incubated with 10–30 nM fumagillin at 4°C for 2 hours. The data were plotted in double-reciprocal plot to examine the mode of inhibition and to calculate the inhibitory constant (*K*i). Kinetic data were estimated using GraphPad Prism version 6 (GraphPad Software, San Diego, USA). Experiments were repeated three times with triplicate replicates per experiment and kinetic values were represented as the means ± SD for independent three assays.

## SUPPLEMENTAL MATERIAL

Supplemental file 1 aac.00560-23-s0001.pdf Fig. S1 to S5.

ASM does not own the copyrights to Supplemental Material that may be linked to, or accessed through, an article. The authors have granted ASM a non-exclusive, world-wide license to publish the Supplemental Material files. Please contact the corresponding author directly for reuse.
