# Supplementary material for: Fumagillin inhibits growth of the enteric protozoan parasite Entamoeba histolytica by covalently binding to and selectively inhibiting methionine aminopeptidase 2
Source: Antimicrob Agents Chemother. 2023 Oct 24;67(11):e00560-23. doi: 10.1128/aac.00560-23 (PMC10648944; doi:10.1128/aac.00560-23)
Supplement: Supplemental file 1 — Fig. S1 to S5. [file aac.00560-23-s0001.pdf]

Supplemental Fig. 1

A

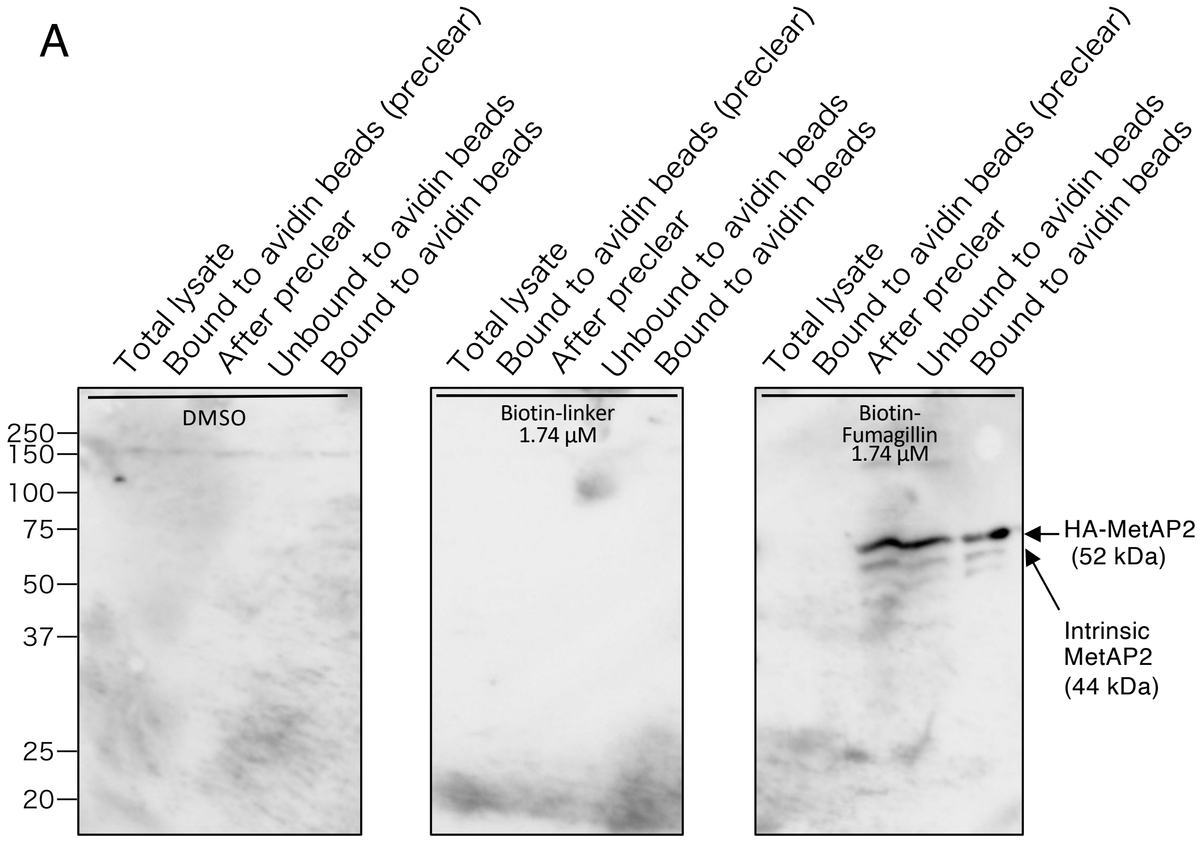

Detection: Avidin-HRP

B

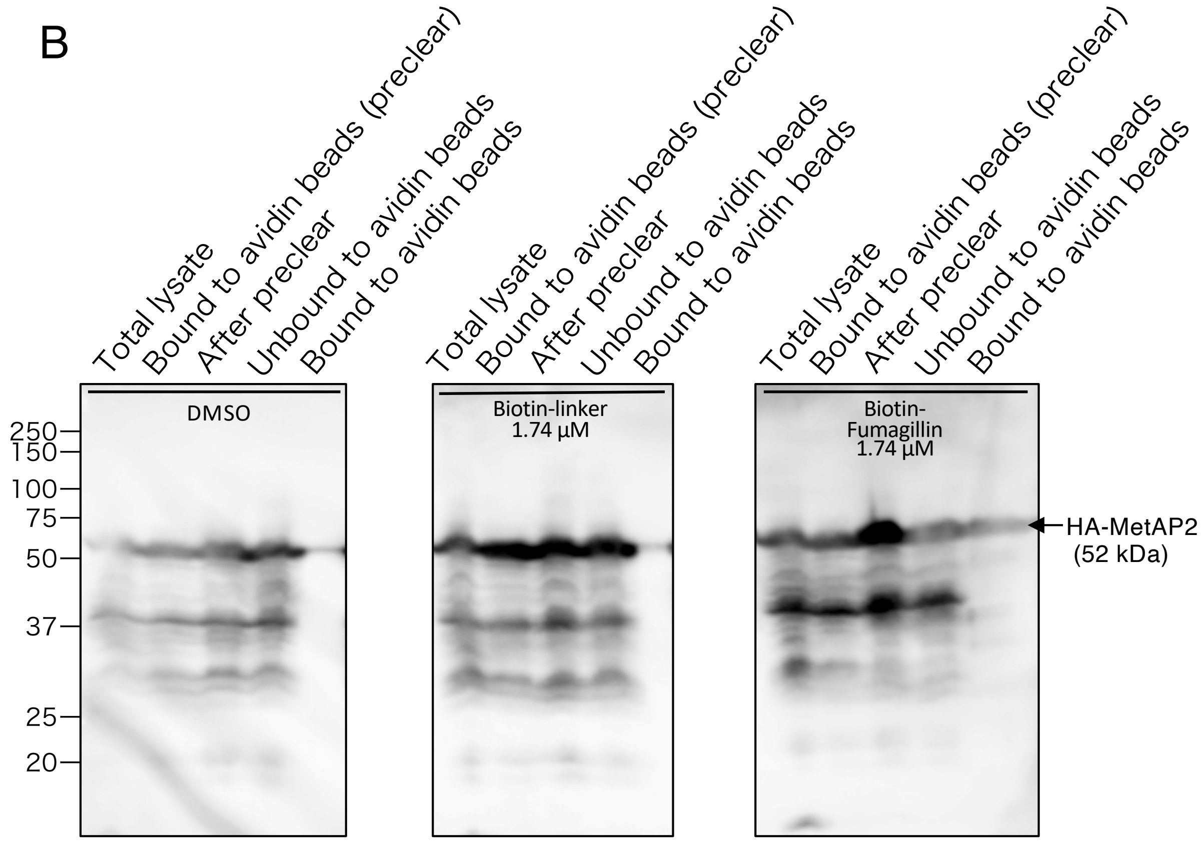

Detection: anti-HA antibody

Fig. S1. HA-EhMetAP2 and intrinsic EhMetAP2 were covalently bound to biotin-fumagillin

(A) Detection of fumagillin-bound proteins from lysates of HA-EhMetAP2-expressing strain. The samples that were affinity-purified from lysates of HA-EhMetAP2 expressing strain with biotinylated fumagillin, biotin-linker, or DMSO only were subjected to SDS-PAGE and detection using Avidin-HRP. The samples obtained at each step of affinity purification ["Total lysate", "Bound to avidin beads (preclear)", "After preclear", "Unbound to avidin beads", and "Bound to avidin beads"] were also subjected to analysis, together with the purified samples. The pull-down experiments were performed as described in Materials and Methods.

(B) Immunoblot analysis of the samples analyzed in (A). HA-EhAP2 was detected with anti-HA antibody.

Supplementary Figure 2

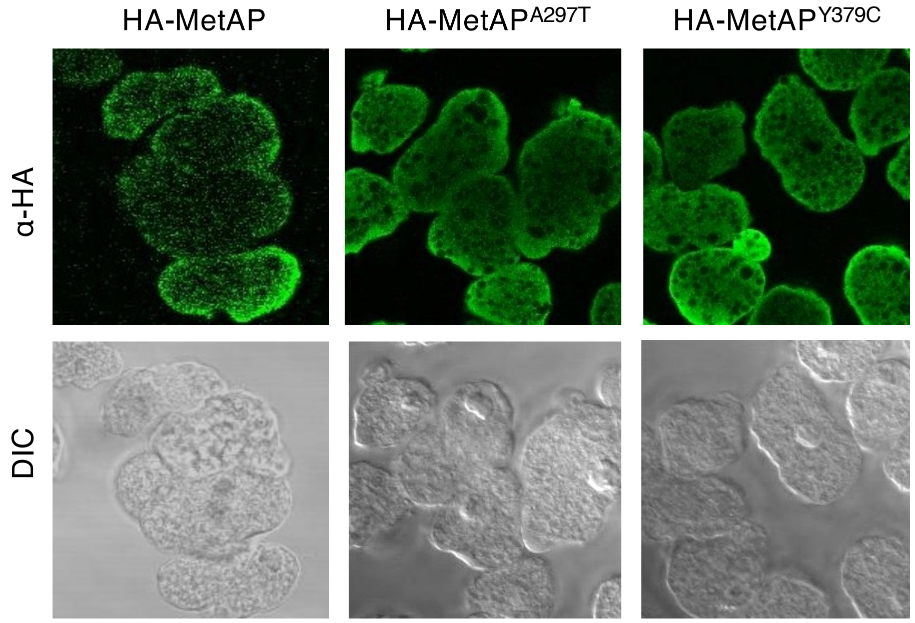

Fig. S2. Immunofluorescence imaging of HA-EhMetAP2, HA-EhMetAP2^{A297T}, HA-EhMetAP2^{Y379C} in *E. histolytica* transformant strains with anti-HA antibody.

Supplementary Figure 3

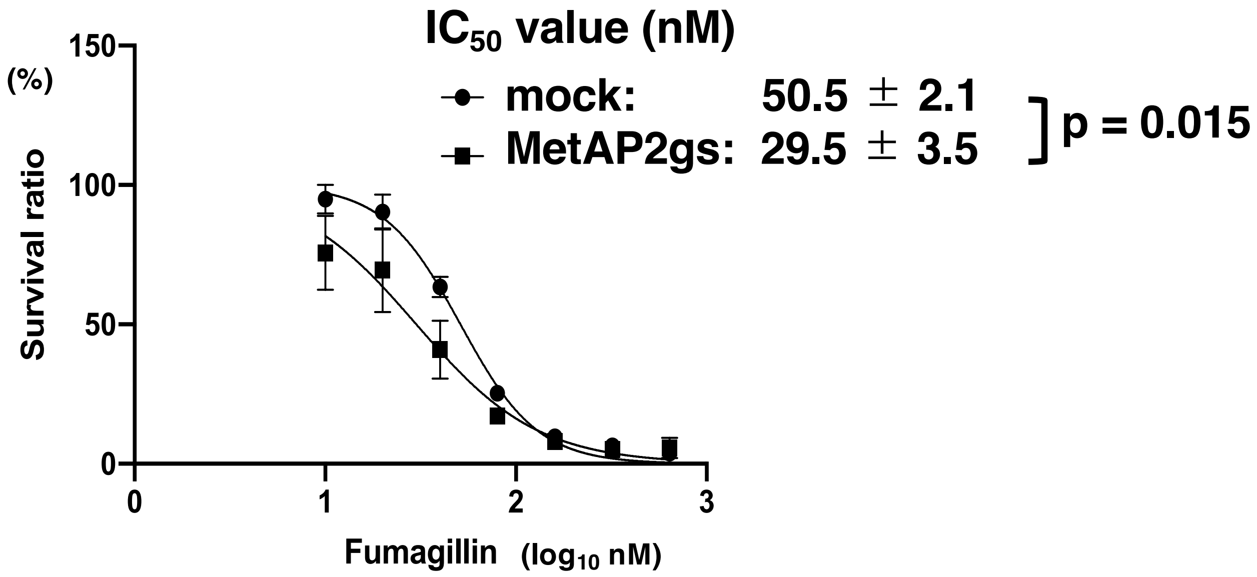

Fig. S3. Susceptivity of *EhMetAP2* gene silenced strain against fumagillin. Growth inhibition of newly transformed EhMetAP2gs and mock control strains by fumagillin. The percentage of live amebae after cultivation with fumagillin at various concentrations for 48 hrs, as estimated by WST-1 assay, is shown.

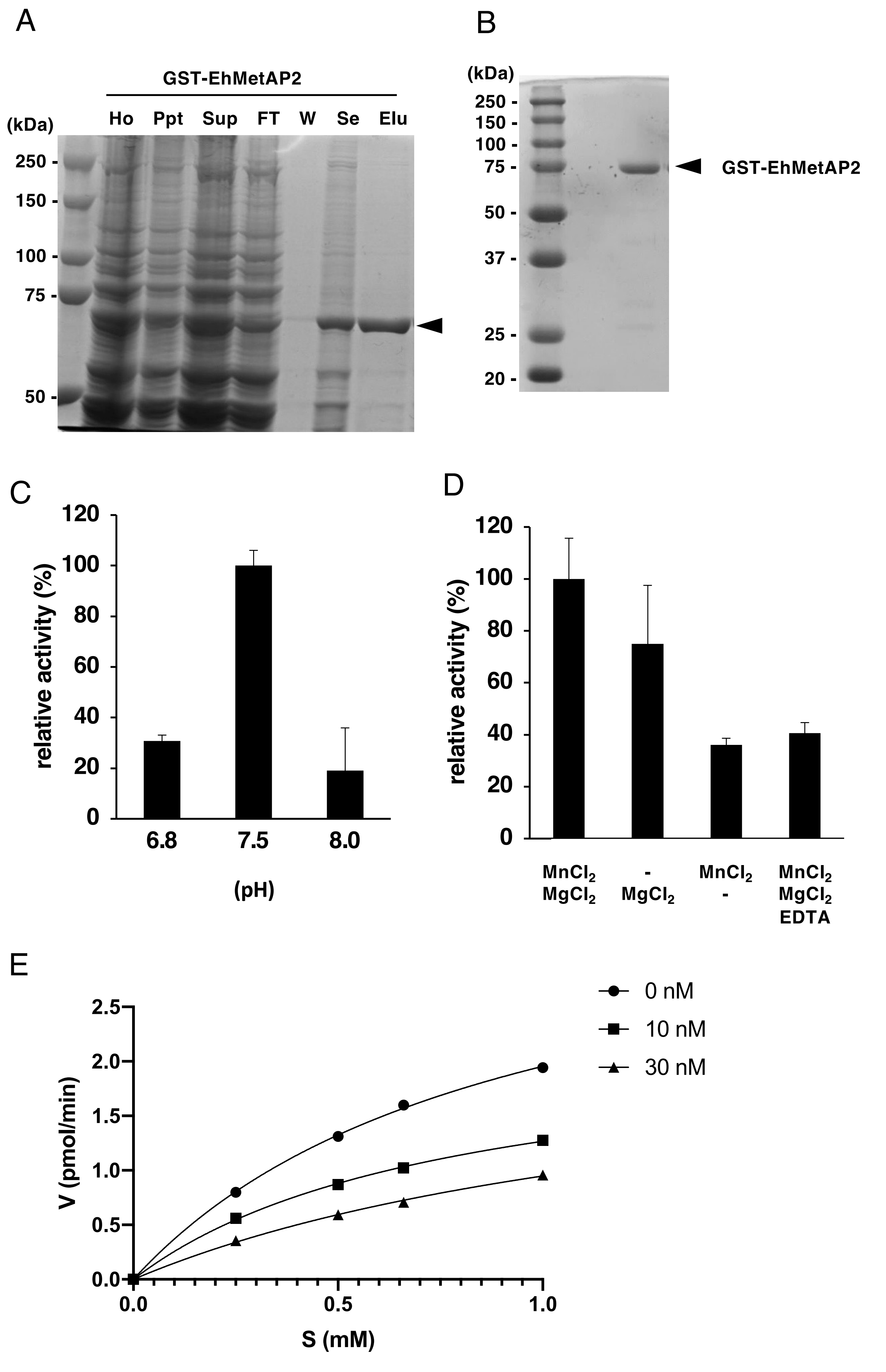
A
GST-EhMetAP2
(kDa)
Ho Ppt Sup FT W Se Elu
250
150
100
75
50
B
(kDa)
250
150
100
75
50
37
25
20
GST-EhMetAP2
C
relative activity (%)
120
100
80
60
40
20
0
6.8 7.5 8.0
(pH)
D
relative activity (%)
120
100
80
60
40
20
0
$MnCl_2$ $MgCl_2$
- $MgCl_2$
$MnCl_2$ -
$MnCl_2$ $MgCl_2$ EDTA
E
0 nM
10 nM
30 nM
V (pmol/min)
2.5
2.0
1.5
1.0
0.5
0.0
0.0 0.5 1.0
S (mM)

Fig. S4. Purification of recombinant EhMetAP2 from yeast expression system and enzyme assay

(A) Expression of GST-EhMetAP2 was induced by the addition of 5 μM $CuSO_4$ and GST-EhMetAP was purified from total yeast lysate with GST-sepharose. GST-EhMetAP2 was eluted with reduced glutathione. Ho: total lysate, Ppt: pellet fraction after centrifuge, Sup: supernatant fraction after centrifuge, FT, flow-through after GST-sepharose purification, W: final wash supernatant, Se: glutathione-eluted GST-sepharose, Elu: final fraction of glutathione-eluted EhMetAP.

(B) Purified GST-EhMetAP Wild type. Ten μl of samples were electrophoresed.

(C) Optimum pH studies showing a pH optimum of 7.5. The optimum pH for the EhMetAP activity against the fluorogenic substrate L-methionine 4-methylcoumaryl-7-amide (Met-MCA) was pH 7.5.

(D) Effect of metal ions on enzymatic activity. $MgCl_2$ is essential for the enzymatic activity and $MnCl_2$ has additive role.

(E) Michaelis-Menten plots of GST-MetAP2 in the presence of 10 (black squares) and 30 (black triangles) nM fumagillin.

Supplementary Figure 5

A

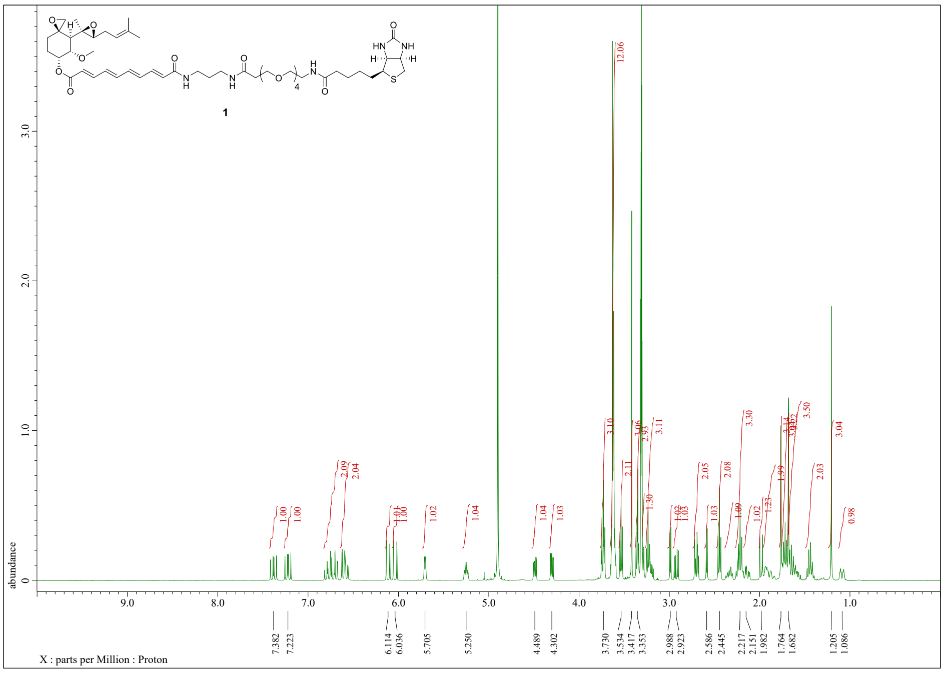

B

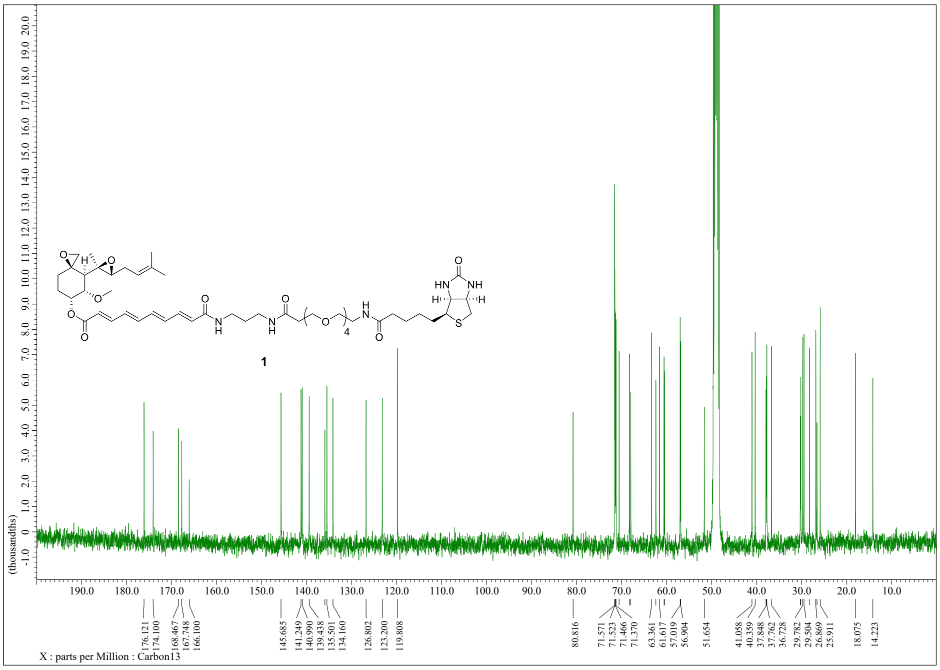

Fig. S5. NMR analysis of biotinylated fumagillin.

(A)^{1}H NMR (400 MHz, CD_3OD) spectrum of biotinylated fumagillin.

(B) $^{13}C\{^{1}H\}$ NMR (100 MHz, CD_3OD) spectrum of biotinylated fumagillin.
